# Facile Strategy for Mass Production of Pt Catalysts for Polymer Electrolyte Membrane Fuel Cells Using Low-Energy Electron Beam

**DOI:** 10.3390/nano10112216

**Published:** 2020-11-06

**Authors:** Jongmin Shin, Jiho Min, Youngjin Kim, Jin Hee Lee, Geunseok Chai, Namgee Jung

**Affiliations:** 1 Graduate School of Energy Science and Technology (GEST), Chungnam National University, 99 Daehak-ro, Yuseong-gu, Daejeon 34134, Korea; ddoraejm@gmail.com (J.S.); mjh9780@naver.com (J.M.); yoongjin123@naver.com (Y.K.); 2 KORENS RTX, 76-32, Ibam-gil, Duma-myeon, Gyeryong-si, Chungcheongnam-do 32842, Korea; 3 Center for Environment & Sustainable Resources, Korea Research Institute of Chemical Technology, Daejeon 34114, Korea; leejh@krict.re.kr

**Keywords:** electron beam, Pt catalyst, fuel cell, mass production, particle size, dispersion, radicals

## Abstract

There are so many variables affecting the large-scale chemical synthesis of nanoparticles that mass production remains a challenge. Here, using a high-efficiency compact electron beam generator irradiating a low-energy electron beam, we fabricate carbon-supported Pt nanoparticles (Pt/C) in an open chamber to present the applicability of an electron beam to the mass production of metal nanocatalysts for polymer electrolyte membrane fuel cells (PEMFCs). The amount of dispersants (glycerol) and radical scavengers (isopropyl alcohol, IPA), the most important factors in the electron beam-induced fabrication process, is systematically controlled to find the conditions for the synthesis of the particle structure suitable for PEMFC applications. Furthermore, the effects of the structural changes on the electrochemical properties of the catalysts are thoroughly investigated. Through in-depth studies, it is clearly revealed that while dispersants control the nucleation step of monomers affecting the degree of dispersion of nanoparticles, radical scavengers with strong oxidizing power have an effect on the particle growth rate. Therefore, this study is expected to present the applicability of low-energy electron beam to the mass production of metal nanocatalysts for PEMFCs, and to provide insights into the fabrication of nanoparticles using low-energy electron beams.

## 1. Introduction

Polymer electrolyte membrane fuel cells (PEMFCs) are attracting much attention as one of the eco-friendly energy conversion devices that directly convert chemical energy into electrical energy without emitting pollutants [1,2,3]. Owing to the use of Pt as a catalyst, PEMFCs exhibit high performance even at a low operating temperature of 60–80 °C compared to other types of fuel cells. However, despite extensive studies on the development of Pt-based catalysts for PEMFCs, in the past decades, most researchers have focused mainly on chemical synthetic methods such as impregnation and reduction methods and polyol-based synthesis. Definitely, there are few reports of successfully commercialized Pt-based catalysts for PEMFCs since the chemical synthesis methods have the following fatal drawbacks [4,5,6,7,8]. In general, the chemical reaction in a reactor takes a long time, and an additional heat or acid treatment process is necessary to remove organic residues and surfactants on the surface of nanoparticles. Particularly, there are so many variables affecting large-scale synthesis that it is very difficult to establish optimal synthesis conditions for mass production of nanocatalysts. Nevertheless, the demand for mass production and commercialization of highly active Pt-based catalysts in the industry has urged researchers to explore new strategies to overcome the limitations of the conventional chemical processes [9,10,11,12].

As an alternative, physical deposition methods using a sputtering system and plasma have been studied to fabricate metal nanoparticles. For instance, Pt or PtNi alloy nanoparticles can be deposited directly onto carbon supports through a sputtering process using polyethylene glycol (PEG) as a liquid substrate [13]. In addition, by controlling the operating conditions, it is possible to produce large amounts of nanoparticles of several nanometers [14,15,16,17]. Therefore, these physical deposition methods have the advantage of being easily scalable for mass production of nanoparticles. However, it is difficult to make nanoparticles with uniform size, and extremely careful treatments such as high-vacuum and evaporation processes in the chamber are still required. Therefore, developing a facile strategy for mass production of nanoparticles remains a challenge.

**Figure 1:**
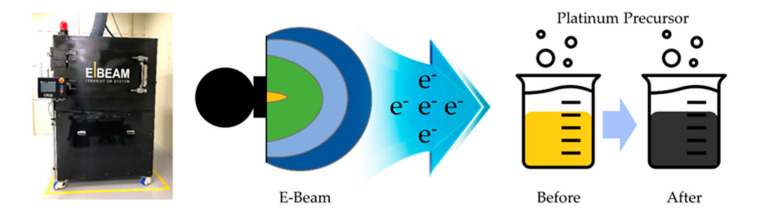
Photograph of the compact electron beam (E-Beam) generator used and schematic illustration showing the reduction of Pt precursors by irradiation of a low-energy electron beam.

On the other hand, as shown in Figure 1, unlike the conventional chemical methods using strong reducing agents for synthesizing metal nanoparticles, hydrated electrons (e_aq_^−^), which are formed in water even at ambient pressure and temperature by direct irradiation of a low-energy electron beam (0.2 MeV), can serve as an extremely powerful reducing agent. The electron beam is accelerated close to the speed of light in the electron beam generator, and the hydrated electrons generated by water decomposition can reduce the metal cation to form metal nanoparticles within a few seconds or minutes. Therefore, it is worthwhile developing an electron beam generator that can be utilized practically for mass production of metal nanoparticles, as an alternative to the traditional chemical processes [18,19,20,21]. In addition, the new nanoparticle fabrication technology using a compact electron beam generator has many advantages over previous studies using other accelerated high-energy radiation sources available only in closed chambers and electron beams that irradiate the local position of nanoparticles for in situ observation of their structural change in transmission electron microscope (TEM) [22,23,24,25,26].

In terms of reaction mechanism, it is well known that by electron beam irradiation, not only hydrated electrons but also reactive radicals (H· and OH·) and other chemical species (H_3_O^+^, H_2_, and H_2_O_2_) are produced simultaneously. However, among them, OH· radicals as a strong oxidizing agent are expected to rapidly oxidize the reduced metal nanoparticles and prevent the growth of the particles [27,28,29]. Accordingly, it is very important to develop a key technology to properly modulate the concentration of reactive radicals during the nanoparticle fabrication process since the application of the nanoparticles to PEMFCs requires a small size of 2–5 nm [30,31,32]. Furthermore, it is necessary to control the dispersion of nanoparticles by selecting the type and amount of dispersants.

Therefore, in this study, a high-efficiency compact electron beam generator irradiating a low-energy (0.2 MeV) electron beam is used to fabricate carbon-supported Pt nanoparticles (Pt/C) in an open chamber. In particular, to find the optimum conditions for particle size and dispersion for PEMFC applications, the structural changes of Pt catalysts depending on the amount of dispersants (glycerol) and radical scavengers (isopropyl alcohol, IPA) are investigated. Furthermore, the effects of the structural changes on the electrochemical properties of the catalysts, including the oxygen reduction reaction (ORR) activity, are thoroughly studied. Through in-depth studies, the roles of radical scavengers and dispersants, which are the key factors in the fabrication process of Pt nanoparticles using a low-energy electron beam, and the importance of their combination are clearly revealed. Therefore, this study will secure a basic but essential database for fabricating single metal and metal alloy nanoparticles with a low-energy electron beam, and provide a breakthrough especially in the development of technology for mass production and commercialization of nanocatalysts for PEMFCs.

## 2. Materials and Methods

### 2.1. Materials

The reagents for the synthesis were used without purification. H_2_PtCl_6_ xH_2_O (10 wt %, T&I Chem, Incheon, Korea) as a Pt precursor, glycerol (99%, DAEJUNG, Siheung-si, Korea) as a dispersant, and isopropyl alcohol (99.5%, IPA, SAMCHUN, Seoul, Korea) as a radical scavenger, and KETJEN BLACK (KB300J, Carbon Black, LION, Tokyo, Japan) as a support were purchased, and deionized (D.I.) water obtained from a tertiary distilled water generator (Angstroms Co., Sejong, Korea) was used as a solvent.

### 2.2. Synthesis of Pt/C Catalyst

Carbon-supported Pt nanoparticles (Pt/C) were synthesized using a low-energy electron beam (0.2 MeV) to reduce Pt precursors. To clearly understand the role of dispersants and scavengers during the electron beam-induced process, the catalysts were prepared with various combinations of dispersant (the molar ratio (G) of glycerol to Pt, G = 100, 200, and 300) and scavenger (IPA = 0, 50, and 200 mL). First of all, 4 g of carbon black was mixed well in 3500 mL D.I. water with appropriate amounts of glycerol and IPA using a tip-type sonicator (20 kHz) for 10 min, and 40 mL of 10 wt % H_2_PtCl_6_ xH_2_O was added to the solution. The well-dispersed solution was then transferred to a separate vessel for the electron beam irradiation in a compact electron beam generator. While the solution was continuously stirred, an electron beam of 0.2 MeV was irradiated for 20 min. After finishing the reaction, the solution was filtered and washed by a copious amount of D.I. water and ethanol (95%, DAEJUNG, Siheung-si, Korea). Finally, the filtered catalyst powders (Pt/C) were then dried at 35 °C in a forced circulation oven (OF-12, JEIO TECH, Seoul, Korea). The prepared samples were designated G(α)-IPA(β) according to the combination of glycerol and IPA initially added to the solution. In G(α)-IPA(β), α and β represent the G value and the amount of IPA, respectively.

### 2.3. Characterizations

TEM (Tecnai G2 F30 S-TWIN, FEI, Hillsboro, OR, USA) analysis was conducted to confirm the particle size and dispersion of synthesized Pt/C catalysts. The crystal structures of Pt nanoparitcles were analyzed using a X-ray diffractometer (XRD, SmartLab, Rigaku, Tokyo, Japan), and for Pt (220) peaks in the XRD data, we used the Scherrer equation [33] to determine the crystallite sizes of the samples, and compared them with the average sizes of Pt nanoparticles obtained from the TEM images. In addition, thermogravimetric analysis (TGA, TGA8000, Woodbridge, ON, USA) was performed in an air atmosphere up to 800 ℃ with a heating rate of 10 °C/min. All electrochemical measurements were tested in a standard three-electrode system using a rotating disk electrode (RDE) with a glassy carbon electrode (GC, 0.196 cm^2^), Ag/AgCl, Pt wire as the working reference, and counter electrodes, respectively. All potential values were represented versus the reversible hydrogen electrode (RHE). The catalyst ink slurry was prepared by mixing 5 mg of Pt/C catalyst, Nafion ionomer (5 wt % in lower aliphatic alcohols and water, Sigma-Aldrich Inc., St. Louis, MO, USA), and IPA (Sigma-Aldrich, 99.5%) in a vial through ultrasonication for 10 min. A drop of catalyst ink was coated on the GC electrode and then dried at room temperature. To investigate the electrochemical properties of the catalysts, cyclic voltammograms (CVs) were measured by cycling the potential between 0.05 and 1.05 V (vs. RHE) at a scan rate of 20 mV s^−1^ in an N_2_-saturated 0.1 M HClO_4_ (Sigma-Aldrich, 70%), and the ORR activities were evaluated with the rotation speed of 1600 rpm at a scan rate of 5 mV s^−1^ in an O_2_-saturated 0.1 M HClO_4_. For comparison, a commercially available 46.7 wt % Pt/C (purchased from Tanaka, TKK, Tokyo, Japan) catalyst, as a chemically synthesized Pt catalyst, was additionally tested.

## 3. Results and Discussion

For the fabrication of metal nanoparticles using an electron beam, polyvinylpyrrolidone (PVP), polyvinyl alcohol (PVA), ethylene glycol (EG), and glycerol, which are usually used in conventional chemical synthesis, can serve as a dispersant since they are generally known to have an effect on the distribution, size, and structure of nanoparticles [34,35,36,37,38,39]. In particular, polyhydric alcohols such as glycerol and EG are expected to have the potential to play a role as a dispersant and a radical scavenger at the same time during the nanoparticle synthesis. Therefore, glycerol can be one of the suitable candidates as a dispersant for the electron beam process. However, since systematic control of reactive radicals generated during the electron beam irradiation becomes more important during the fabrication process, structural changes such as the size and distribution of Pt nanoparticles by addition of IPA as a powerful radical scavenger with glycerol (dispersant) were carefully observed. Furthermore, the effects of the combination of dispersants and radical scavengers on the electrochemical properties of Pt catalysts were thoroughly investigated. By finding the optimum conditions under the irradiation of a low-energy electron beam, the fabrication process could be considerably simplified compared to the conventional chemical synthesis due to no surfactants and reducing agents being used, and enabled the mass production of Pt/C catalysts (~8 g/batch) at room temperature. In addition, as shown in Table 1 and Figure S1 (TGA data), it was confirmed that the synthesized Pt/C catalysts showed acceptable and similar Pt content (45.3–49.7 wt % Pt/C) when targeting 50 wt % Pt/C. Since the weight and atomic ratios of Pt to C for the catalysts can be calculated from the TGA results, they were also summarized in Table S1 [40,41].

**Figure 2:**
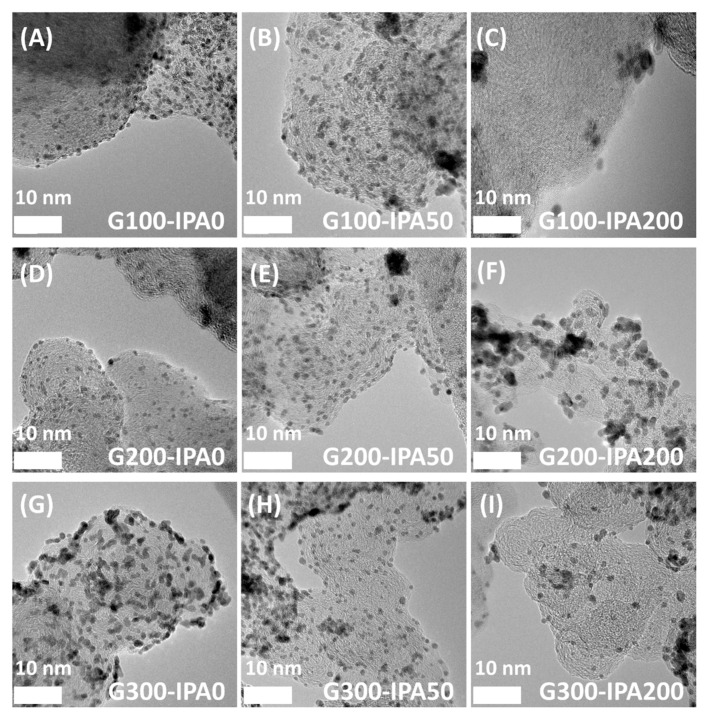
TEM images of (**A**) G100-IPA0, (**B**) G100-IPA50, (**C**) G100-IPA200, (**D**) G200-IPA0, (**E**) G200-IPA50, (**F**) G200-IPA200, (**G**) G300-IPA0, (**H**) G300-IPA50, and (**I**) G300-IPA200 samples.

Figure 2 exhibits the TEM images showing the structural changes of Pt nanoparticles according to the content of glycerol and IPA used as a dispersant and a radical scavenger, respectively, under the condition for generating hydrated electrons for reduction of Pt precursors without disturbing the accelerated electron flow in the reaction vessel. In addition, from the TEM images in Figure 2, the particle size distributions and average particle sizes were determined (Figure S2). First of all, in the case of the G100 (the molar ratio of glycerol to Pt = 100) samples, when IPA was used as much as 0 (IPA0) or 50 mL (IPA50), small particles were well distributed on carbon supports. However, when the amount of IPA significantly increased to 200 mL (IPA200), the particle dispersion deteriorated and the Pt particles severely agglomerated, resulting in a significant decrease in the particle density on the surface of the carbon support. Under the condition of G200 (the molar ratio of glycerol to Pt = 200), even if the IPA content increased, high dispersion of Pt nanoparticles was generally maintained while the particles gradually agglomerated. On the other hand, in the case of the G300 (the molar ratio of glycerol to Pt = 300) samples, particle agglomeration occurred even under the IPA0 condition (without IPA), resulting in a non-uniform particle size. Moreover, it was confirmed that the phenomenon became more severe as the IPA content increased to 50 (IPA50) and 200 mL (IPA200). Therefore, it can be concluded that if OH· radicals with strong oxidizing power are removed too much by radical scavengers, the nucleation and growth rate of Pt nanoparticles might be considerably affected at the same time.

**Figure 3:**
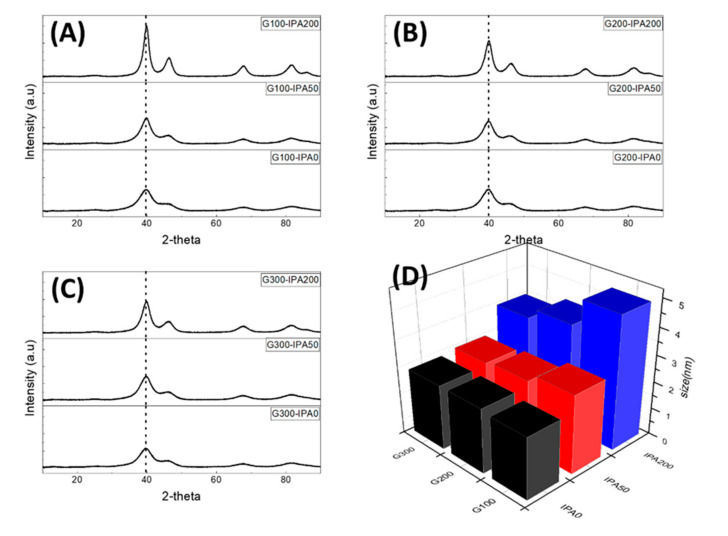
XRD patterns of Pt/C catalysts synthesized using different IPA contents (0, 50, and 200 mL) in fixed amounts of glycerol of (**A**) G100, (**B**) G200, and (**C**) G300. (**D**) Particle sizes of Pt/C catalysts according to the reaction conditions.

Figure 3 shows the results of XRD analysis to compare the difference in the crystal structure and the change in the particle size (nm) of the catalysts according to the amount of dispersants and radical scavengers. As shown in Figure 3A–C, since all catalysts were composed of single Pt, 2 theta degrees of typical Pt nanoparticles were identified at 39.8°, 46.2°, 67.5°, and 81.3° for Pt(111), (200), (220), and (311) facets, respectively [3,42]. However, when the same amount of dispersant (glycerol) was used, the XRD peak intensity increased consistently by increasing the IPA (radical scavenger) content, indicating a result of agglomeration of Pt nanoparticles (increased particle size). Especially, as shown in Figure 3D and Table 2, the smaller the amount of dispersants, the more remarkable the particle (or crystallite) size changes depending on the amount of radical scavenger added. On the other hand, when the amount of dispersants increased, the particle size was not significantly affected by the amount of radical scavengers. Representatively, the G100-IPA0, G100-IPA50, and G100-IPA200 samples had particle sizes of 2.3, 2.9, and 4.9 nm, respectively, showing a tendency that the particle size increases sharply as the amount of IPA increases. In sharp contrast, the G300-IPA0, G300-IPA50, and G300-IPA200 samples indicated particle sizes of 2.4, 2.5, and 3.5 nm, respectively, implying that there is no significant difference in the particle size.

From the XRD analysis result and the TEM image in Figure 2, the roles of dispersant and radical scavenger during the synthesis of Pt nanoparticles using electron beams were more clearly revealed. First of all, while the dispersant basically provides highly dispersed metal ion complexes (monomers) for burst nucleation, it does not effectively remove the radicals with strong oxidizing power. As a result, the reactive radicals generated by electron beams can still prevent the growth of the particle size to an appropriate level. However, as the amount of IPA increases in the same amount of dispersants, the particle size tends to increase, and severe agglomeration occurs in excessive amounts. Therefore, IPA plays an effective role in rapidly removing the radicals that inhibit the growth of Pt nanoparticles. Consequently, it is considered that the addition of an appropriate amount of IPA contributes to uniform particle growth by lowering the surface energy of the nanoparticles.

**Figure 4:**
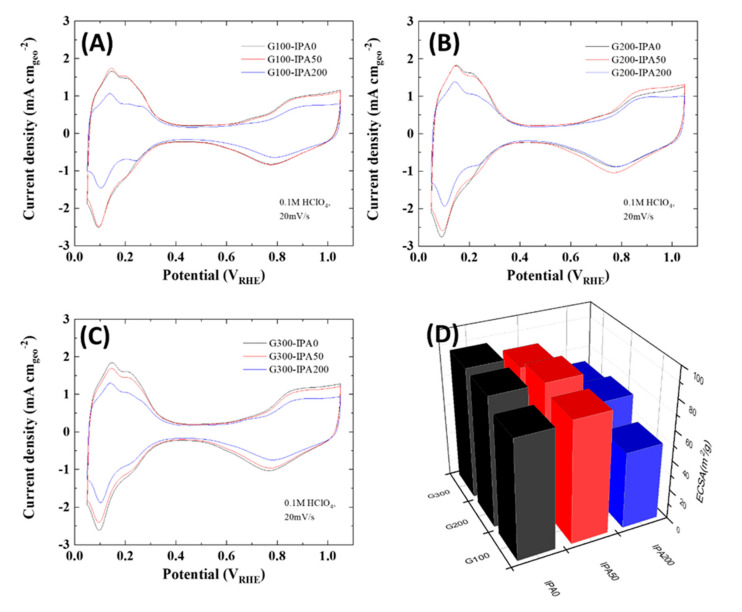
Cyclic voltammograms (CVs) of Pt/C catalysts synthesized using different IPA contents (0, 50, and 200 mL) in fixed amounts of glycerol of (**A**) G100, (**B**) G200, and (**C**) G300. (**D**) ECSAs of Pt/C catalysts according to the reaction conditions.

As shown in Figure 4, the electrochemical properties of synthesized Pt/C catalysts were analyzed using a 3-electrode system, and their cyclic voltammograms (CVs) were first compared to observe the difference in the electrochemical surface areas (ECSAs) according to the changes in the amount of dispersants and scavengers. As shown in Figure 4D, the ECSAs generally decreased by increasing the IPA content in the same amount of dispersant. In particular, the G100 samples, which have relatively small amount of dispersants, showed the largest change in the ECSAs between G100-IPA0 (IPA 0 mL) and G100-IPA200 (IPA 200 mL). On the other hand, the G300 samples exhibited still higher ECSAs compared to the G100 sample even when the IPA content was 200 mL (G300-IPA200). Corresponding to the results of XRD and TEM analysis, it is confirmed that the dispersant induces high dispersion of the particles, while the significant decrease in the amount of radicals by addition of radical scavengers leads to rapid growth of Pt nanoparticles. Consequently, the reduction of the ECSA is attributed mainly to an increase in the amount of radical scavengers. 

**Figure 5:**
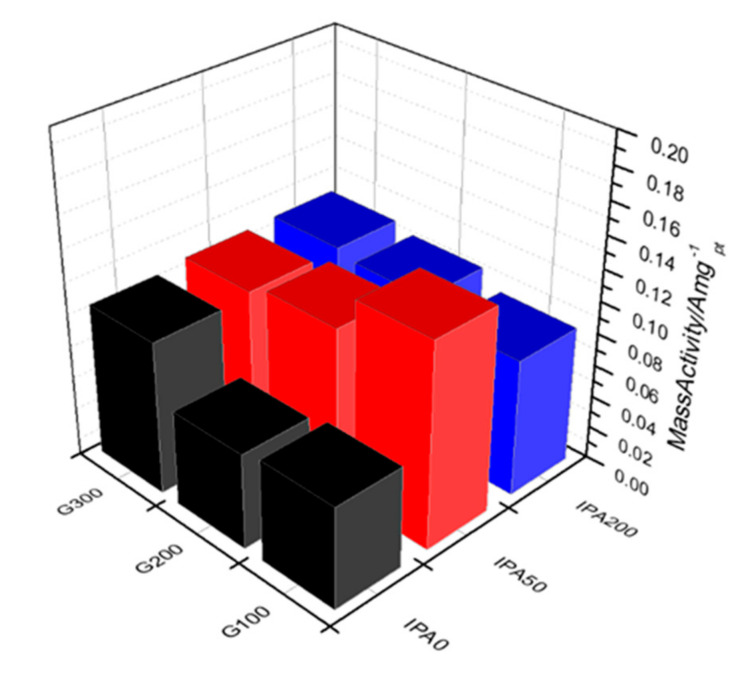
Mass activities for ORR of synthesized Pt/C catalysts.

Finally, for PEMFC application, the ORR activities of Pt nanoparticles depending on the amount of dispersants and radical scavengers were evaluated as shown in Figure 5 and Figure S3, and all electrochemical properties of the catalysts including half-wave potential, mass activity, and ECSA were summarized in Table 3. The mass activity, an indicator of the catalytic activity per weight of Pt used, was calculated at 0.9 V, where the mass transport of O_2_ molecules has little effect during ORR. As expected, the mass activity for the ORR was determined by the catalyst particle size and dispersion, which changed according to the difference in the relative amounts of dispersants and scavengers. For instance, the G100-IPA0 and G200-IPA0 samples, which were very small in particle size and highly dispersed, exhibited remarkably low mass activities despite showing relatively large ECSAs compared to other Pt catalysts. This is consistent with the theoretical calculation results that the smaller the Pt nanoparticles, the narrower the *d* band width, resulting in a decrease in ORR activity due to the strong oxygen binding energy [43,44]. On the other hand, the G100-IPA50 sample showed the highest mass activity of 0.127 A/mg_Pt_ at 0.9 V_RHE_ and an ORR polarization curve similar to that of the commercially available Pt/C (Figure S4), implying that the highly dispersed Pt particles of ~3 nm size have the most suitable *d* band structure and oxygen binding energy for ORR [44,45]. Therefore, in the fabrication process of metal nanoparticles using a low-energy electron beam, while dispersants control burst nucleation of monomers affecting the degree of dispersion of nanoparticles, radical scavengers with strong oxidizing power have an effect on the particle growth rate. This suggests that an appropriate combination of a dispersant and a radical scavenger is required to produce a metal nanocatalyst having a particle size and a dispersion suitable for an application.

## 4. Conclusions 

In this study, a facile strategy for mass production of carbon-supported Pt nanoparticles for ORR using hydrated electrons (e_aq_^−^) by irradiating a low-energy electron beam was proposed. Pt/C catalysts of 8 g/batch were successfully synthesized using a compact electron beam generator. However, during the electron beam irradiation, since various chemical species such as H, OH, H_3_O^+^, H_2_, and H_2_O_2_, as well as hydrated electrons are simultaneously produced, the dispersion and size of Pt nanoparticles strongly depend on the synthesis conditions. Therefore, to find the most suitable structure of Pt nanoparticles for PEMFC application, the ratio of glycerol (dispersant) and IPA (radical scavenger) was systematically controlled, and the changes in the ORR activities depending on the structure of Pt nanoparticles were thoroughly investigated. Through in-depth studies, it was clearly revealed that while dispersants control the nucleation step of monomers affecting the degree of dispersion of nanoparticles, radical scavengers which can remove reactive radicals oxidizing reduced nanoparticles have an effect on the particle growth rate. Therefore, this study is expected to present the applicability of low-energy electron beam to the mass production of metal nanocatalysts for PEMFC, and to provide important insights into the fabrication of single metal and metal alloy nanoparticles using low-energy electron beams.

## Figures and Tables

**Table 1:** Pt content (wt %) of the prepared Pt/C catalysts (estimated from TGA).

Samples	Pt Content (wt %)
G100-IPA0	47.7
G100-IPA50	45.3
G100-IPA200	46.6
G200-IPA0	47.5
G200-IPA50	47.5
G200-IPA200	49.7
G300-IPA0	48.4
G300-IPA50	48.3
G300-IPA200	49.2

**Table 2:** Particle and crystallite sizes, which were obtained from TEM and XRD analyses, respectively, of the samples synthesized under different conditions.

Samples	Particle Size (nm)	Crystallite Size (nm)
G100-IPA0	2.3	2.0
G100-IPA50	2.9	2.3
G100-IPA200	4.9	3.7
G200-IPA0	2.4	2.0
G200-IPA50	2.6	2.3
G200-IPA200	3.9	3.0
G300-IPA0	2.4	2.1
G300-IPA50	2.5	2.2
G300-IPA200	3.5	2.8

**Table 3:** Electrochemical properties of Pt/C catalysts synthesized using a low-energy electron beam.

Samples	Half-Wave Potential (V)	Mass Activity (A/mg_Pt_ at 0.9 V_RHE_)	ECSA (m^2^/g_Pt_)
G100-IPA0	0.906	0.063	78.7
G100-IPA50	0.926	0.127	80.7
G100-IPA200	0.917	0.085	51.0
G200-IPA0	0.901	0.059	86.1
G200-IPA50	0.924	0.104	86.3
G200-IPA200	0.922	0.095	65.8
G300-IPA0	0.922	0.095	87.4
G300-IPA50	0.920	0.098	80.5
G300-IPA200	0.922	0.099	61.4

## Supplementary Materials

The following are available online at https://www.mdpi.com/2079-4991/10/11/2216/s1, Figure S1: TGA data to estimate the Pt content (wt %) of the prepared Pt/C catalysts; Figure S2: Particle size distributions and average particle sizes of Pt/C catalysts according to the reaction conditions; Figure S3: ORR polarization curves of Pt/C catalysts synthesized using different IPA contents (0, 50, and 200 mL) in fixed amounts of glycerol of (A) G100, (B) G200, and (C) G300 (D) Mass activities of Pt/C catalysts according to the reaction conditions; Figure S4: (A) TEM image and particle size distribution of commercial Pt/C and (B) comparison of ORR polarization curves of commercial Pt/C and G100-IPA50 sample; Table S1: The weight and atomic ratios of Pt to C for the prepared catalysts (calculated from TGA results).
