# Supplementary material for: Facile Strategy for Mass Production of Pt Catalysts for Polymer Electrolyte Membrane Fuel Cells Using Low-Energy Electron Beam
Source: Nanomaterials (Basel). 2020 Nov 6;10(11):2216. doi: 10.3390/nano10112216 (PMC7694981; doi:10.3390/nano10112216)
Supplement: Supplementary file 1 [file nanomaterials-10-02216-s001.pdf]

nanomaterials

Supplementary Materials: Facile Strategy for Mass Production of Pt Catalysts for Polymer Electrolyte Membrane Fuel Cells Using Low-Energy Electron Beam

Jongmin Shin [1,2], Jiho Min [1], Youngjin Kim [1], Jin Hee Lee [3], Geunseok Chai [2,*] and Namgee Jung [1,*]

1 Graduate School of Energy Science and Technology (GEST), Chungnam National University, 99 Daehak-ro, Yuseong-gu, Daejeon 34134, Korea; ddoraejm@gmail.com (J.S.); mjh9780@naver.com (J.M.); yoongjin123@naver.com (Y.K.)
2 KORENS RTX, 76-32, Ibam-gil, Duma-myeon, Gyeryong-si, Chungcheongnam-do 32842, Korea
3 Center for Environment & Sustainable Resources, Korea Research Institute of Chemical Technology, Daejeon 34114, Korea; leejh@krict.re.kr
* Correspondence: gschai@irtx.co.kr (G.C.); njung@cnu.ac.kr (N.J.)

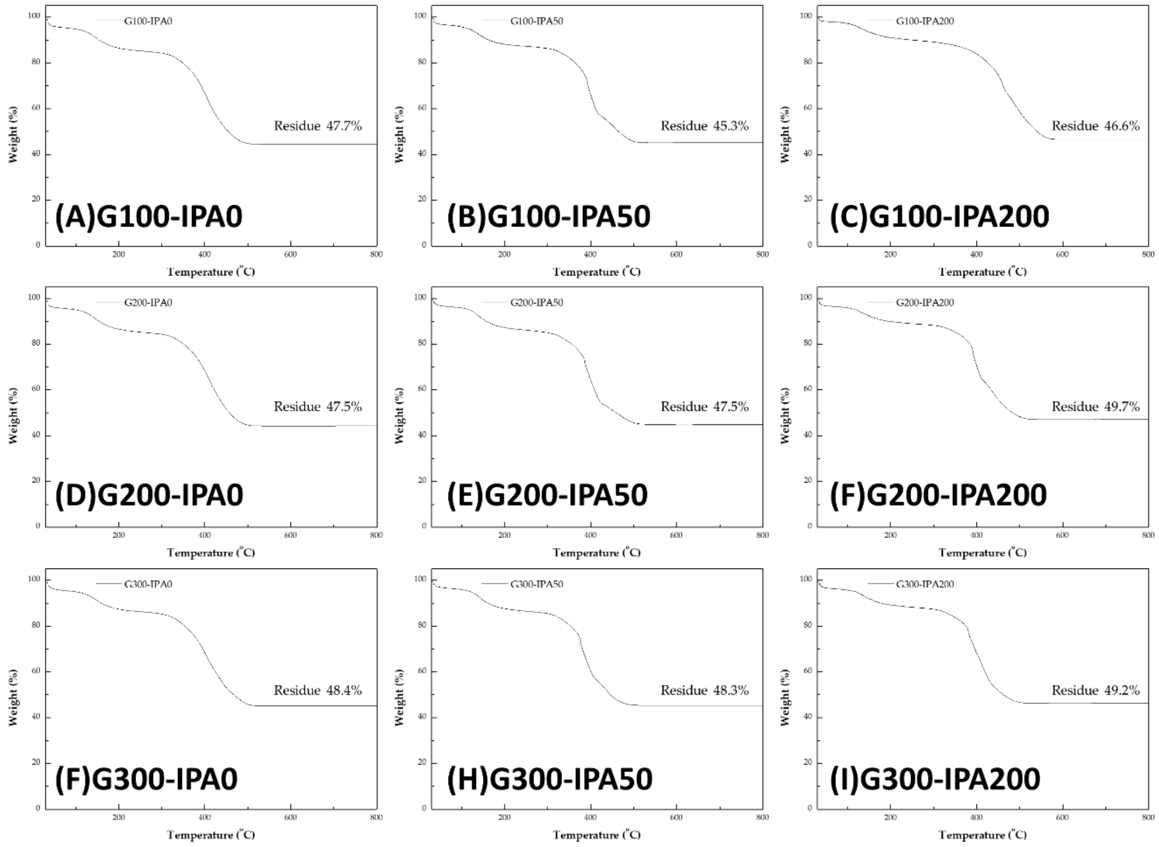

Figure S1. TGA data to estimate the Pt content (wt %) of the prepared Pt/C catalysts.

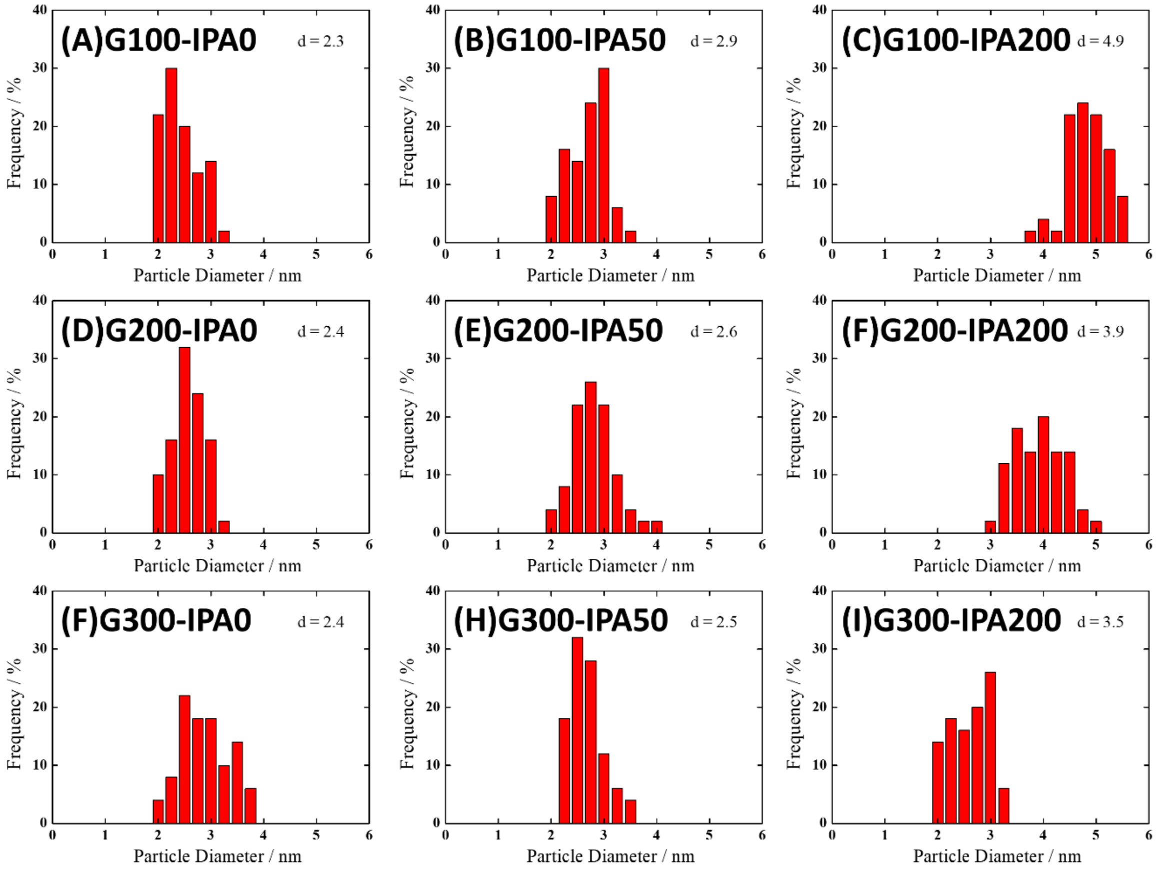

Figure S2. Particle size distributions and average particle sizes of Pt/C catalysts according to the reaction conditions.

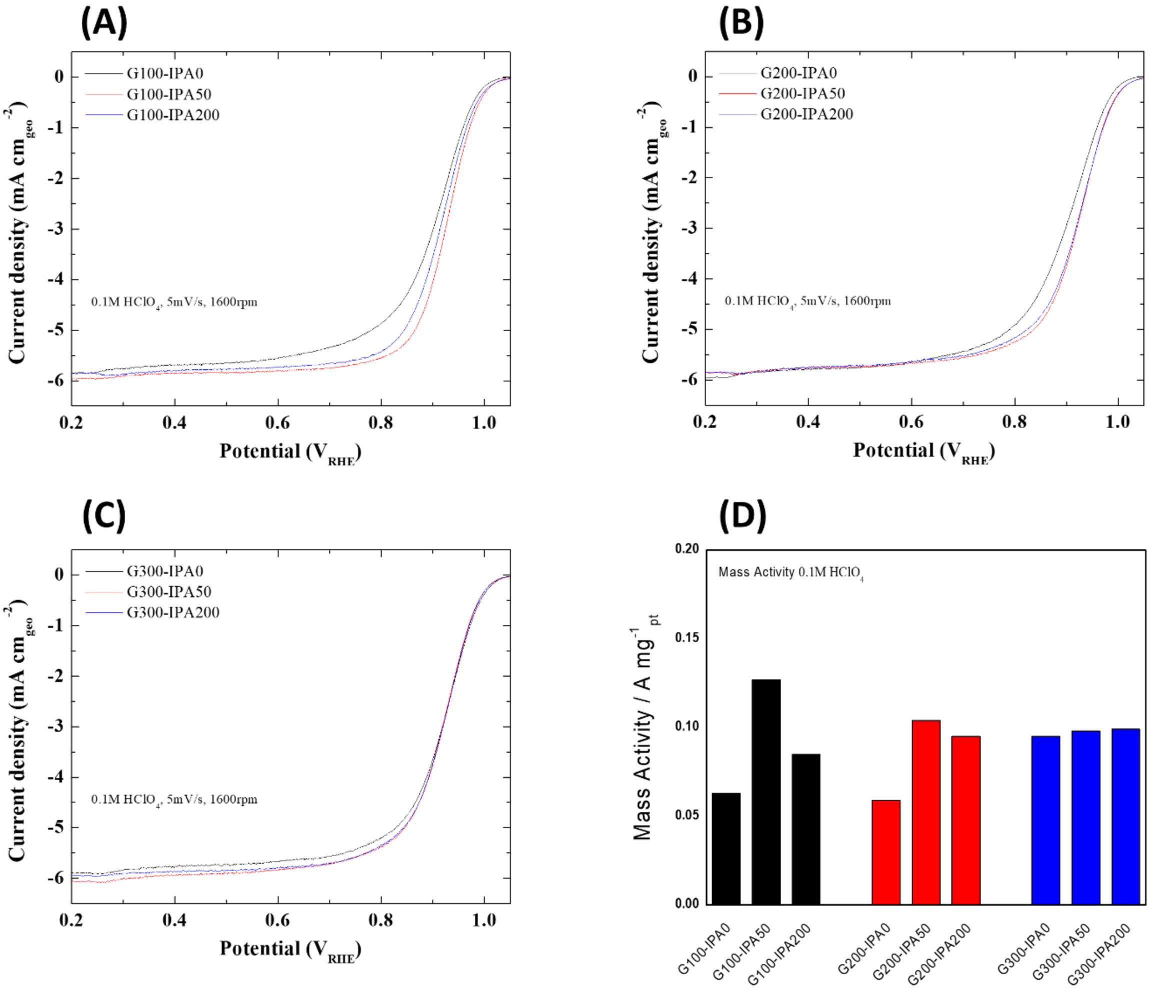

Figure S3. ORR polarization curves of Pt/C catalysts synthesized using different IPA contents (0, 50, and 200 mL) in fixed amounts of glycerol of (A) G100, (B) G200, and (C) G300. (D) Mass activities of Pt/C catalysts according to the reaction conditions.

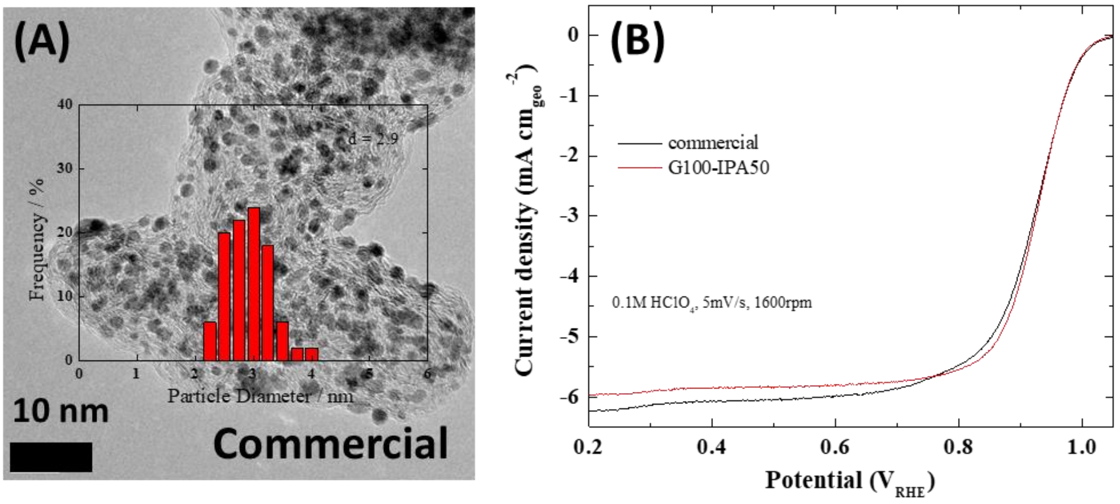

Figure S4. (A) TEM image and particle size distribution of commercial Pt/C and (B) comparison of ORR polarization curves of commercial Pt/C and G100-IPA50 sample.

Table S1. The weight and atomic ratios of Pt to C for the prepared catalysts (calculated from TGA results).

Samples	Weight Ratio	Atomic Ratio
G100-IPA0	0.91	0.06
G100-IPA50	0.83	0.05
G100-IPA200	0.87	0.05
G200-IPA0	0.90	0.06
G200-IPA50	0.90	0.06
G200-IPA200	0.99	0.06
G300-IPA0	0.94	0.06
G300-IPA50	0.93	0.06
G300-IPA200	0.97	0.06
